# Therapies from Thiopeptides

**DOI:** 10.3390/molecules28227579

**Published:** 2023-11-14

**Authors:** Hee-Jong Hwang, Marco A. Ciufolini

**Affiliations:** 1 Department of Chemistry, The University of British Columbia, 2036 Main Mall, Vancouver, BC V6T 1Z1, Canada; hwanghj@anjscience.co.kr; 2 A&J Science, Ltd., 80 Chumbok Ro, Dong Gu, Daegu 41061, Republic of Korea

**Keywords:** antibiotics, *Clostridioides difficile*, c-kit, COVID, kinase inhibitors, micrococcin P1, microtubules, thiopeptides, total synthesis

## Abstract

The first part of this contribution describes solutions that were developed to achieve progressively more efficient syntheses of the thiopeptide natural products, micrococcins P1 and P2 (MP1–MP2), with an eye toward exploring their potential as a source of new antibiotics. Such efforts enabled investigations on the medicinal chemistry of those antibiotics, and inspired the development of the kinase inhibitor, Masitinib^®^, two candidate oncology drugs, and new antibacterial agents. The studies that produced such therapeutic resources are detailed in the second part. True to the theme of this issue, “Organic Synthesis and Medicinal Chemistry: Two Inseparable Partners”, an important message is that the above advances would have never materialized without the support of curiosity-driven, academic synthetic organic chemistry: a beleaguered science that nonetheless has been—and continues to be—instrumental to progress in the biomedical field.

## 1. Introduction

Thiopeptide natural products [1] are sulfur-rich, structurally complex substances that exhibit noteworthy activity against Gram-positive microorganisms [2]. Retrosynthetic considerations unveil numerous difficulties associated with a synthetic attack on their architecture. This has provided much opportunity in the synthetic arena to devise innovative solutions and contribute significant advances in heterocyclic chemistry [3,4,5,6,7,8,9,10,11,12,13,14].

The authors’ own involvement in the field, originally motivated purely by the synthetic challenge of conquering at least some of the thiopeptides, ultimately inspired the development of a number of therapeutic agents, one of which is approved in Europe for certain veterinary applications and is in advanced Phase III clinical trials for a number of human pathologies.

In the spirit of the theme of this Special Issue, “Organic Synthesis and Medicinal Chemistry, Two Inseparable Partners: Recent Advances in Heterocyclic Chemistry”, this contribution recounts the development of key solutions devised in the course of synthetic efforts directed toward micrococcins P1–P2 and thiocillin I, and the translation of these activities into medicinal chemistry campaigns that produced a marketed kinase inhibitor, an antitumor agent about to enter clinical trials, and most recently, two antibacterial clinical candidates that address urgent medical problems. The latter endeavor was launched in response to the ongoing antibiotic crisis [15], namely the emergence of bacterial pathogens that have become resistant to antibiotics—even those of a last resort. It seemed plausible that the antibacterial potency of **1**–**3** could be harnessed to create new weapons against the threat posed by such resistant organisms.

## 2. Synthetic Efforts toward Representative Thiopeptide Antibiotics

**Scheme 1:**
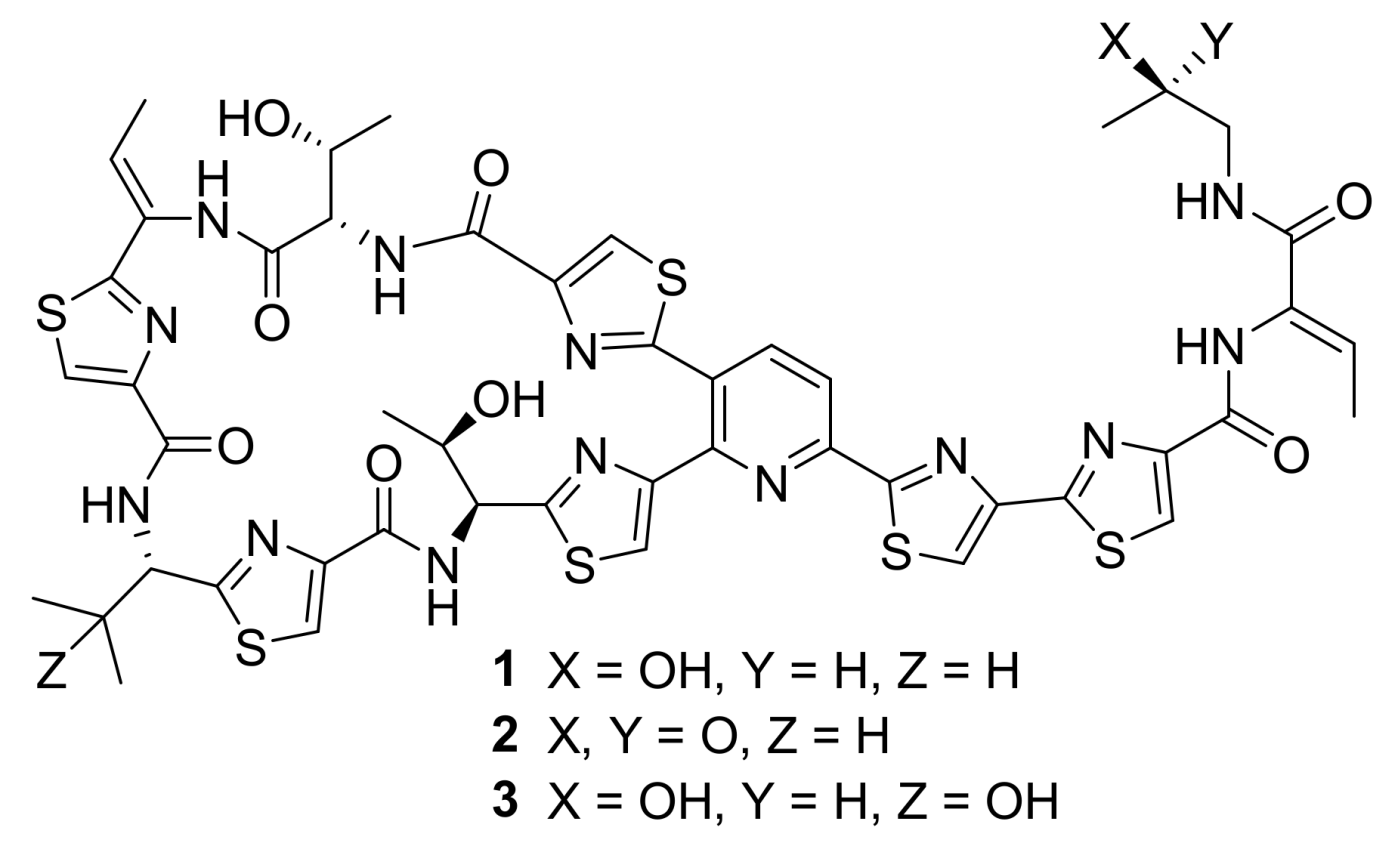
Structures of microccins P1 (**1**), P2 (**2**), and thiocillin I (**3**).

Micrococcins P1, **1**, and P2, **2** [16], and the structurally related thiocillin I, **3** [17] (Scheme 1), are among the simplest thiopeptides possessing valuable antibacterial activity, albeit only toward Gram-positive organisms. While this undoubtedly constitutes a limitation, the U.S. Center for Disease Control and Prevention (CDC) lists several Gram+ bacteria as species of concern [18]. Compounds **1**–**3** thus appeared to be excellent platforms for the development of new antibacterial resources based on thiopeptide motifs.

**Scheme 2:**
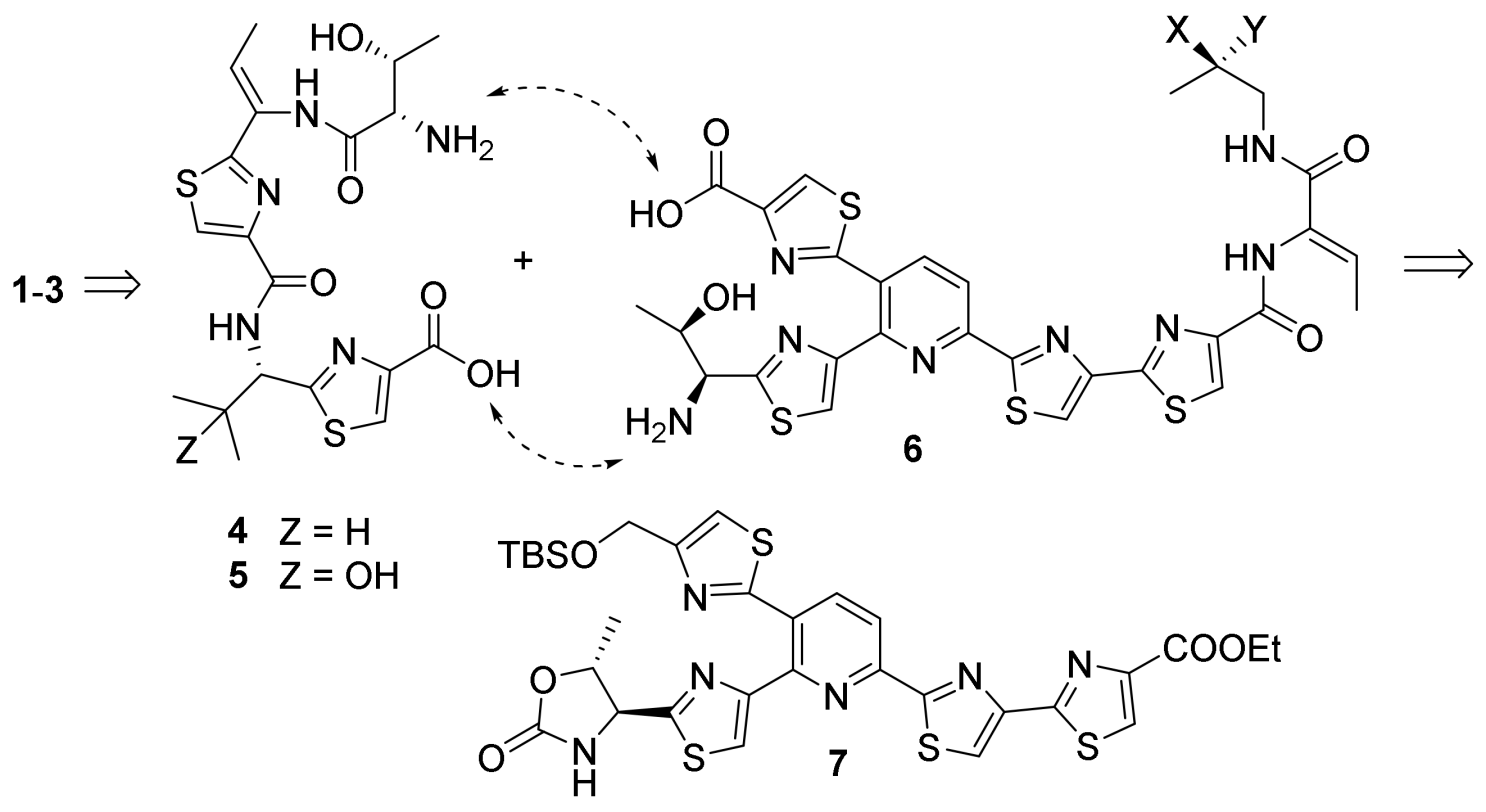
Retrosynthetic logic for **1**–**3**.

The establishment of a good total synthesis was deemed to be essential in light of a possible medicinal chemistry investigation that might lead to clinical candidates, because the natural products do not lend themselves easily to chemical modification or derivatization. Retrosynthetic considerations (Scheme 2) suggested that a critical subgoal would be the assembly of the pyridine-thiazole cluster, **7**, as rapidly, convergently, and efficiently as possible. This ruled in favor of methods other than transition metal-mediated coupling reactions, at least at this juncture. Elaboration of **7** to **6** and merger of the latter with segments **4** or **5** would deliver fully synthetic **1**–**3**. In keeping with such guidelines, this section centers on successive refinements of routes to structures of the type **7**.

**Scheme 3:**
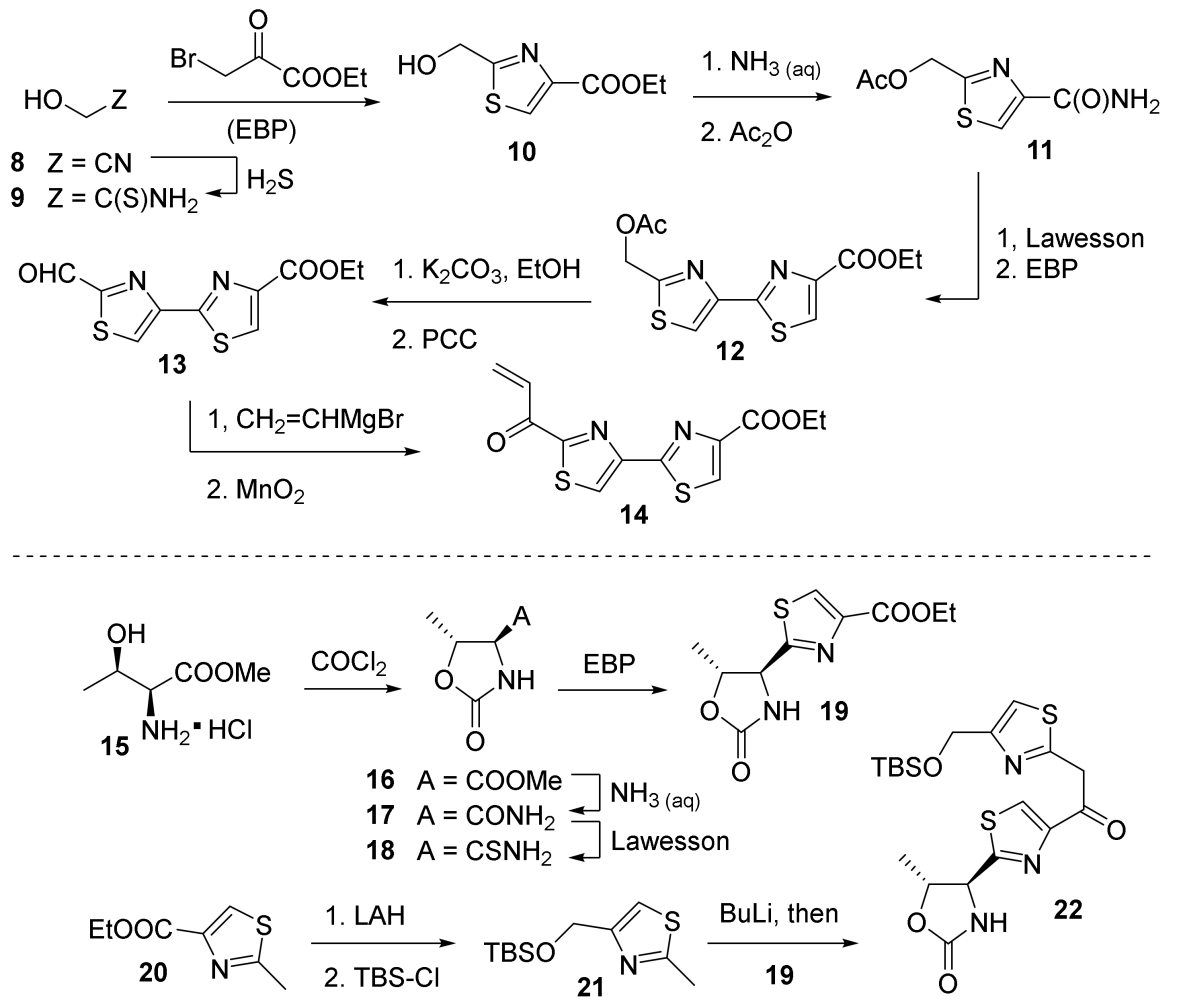
Assembly of fragments **14** and **22**.

An early avenue to **7** [19] started with the assembly of fragments **14** and **22** (Scheme 3) as components of a Hantzsch-type pyridine synthesis [20] that was anticipated to furnish **7**. It is already apparent that the routes to these intermediates are fairly lengthy, especially that to enone **14**. The following two weaknesses are worthy of note. First, the conversion of commercial glycolonitrile **8** to thioamide **9** was inefficient. Second, three equivalents of the anion of **21** were required in the reaction with **19**: one to deprotonate the relatively acidic oxazolidinone NH, one to add to ester carbonyl, and one to deprotonate the emerging **22**, the C-H acidity of which was comparable to that of a 1,3-diketone. However, our objective then was the completion of the total synthesis, so that the search for more direct alternatives was postponed to a more opportune time.

**Scheme 4:**
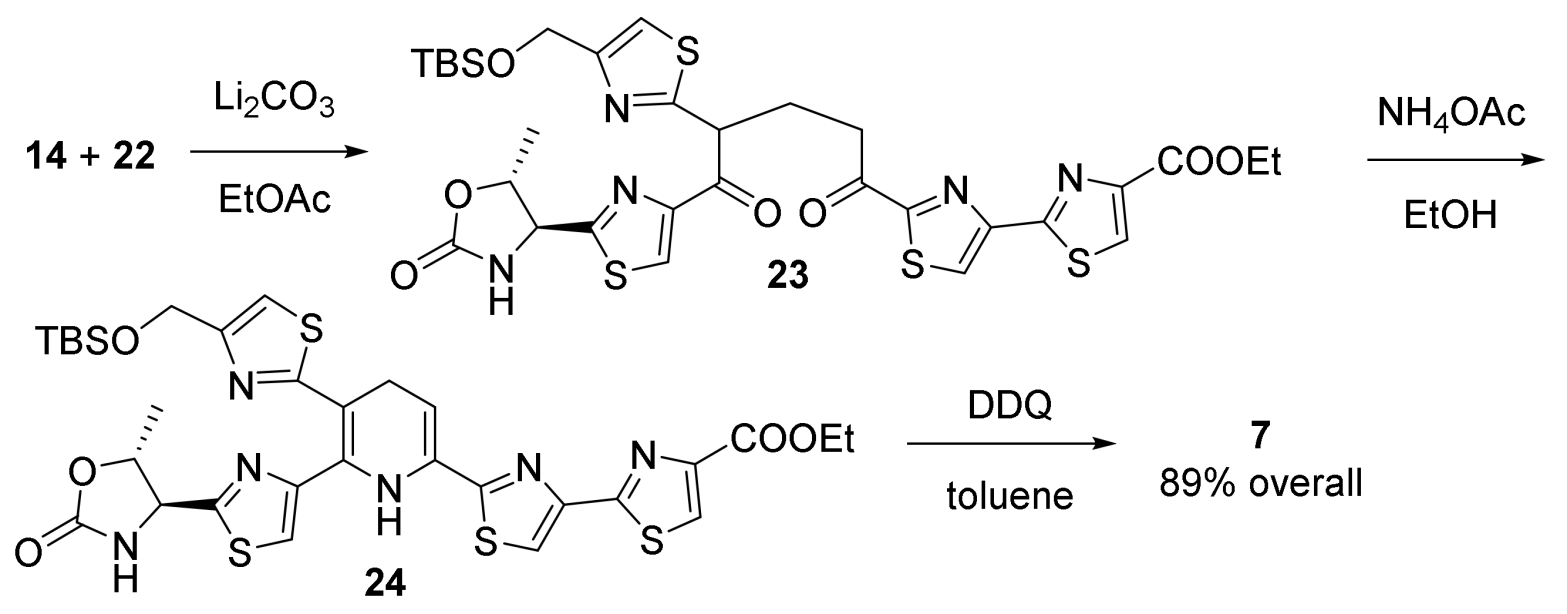
Hatzsch route to pyridine **7**.

The seemingly straightforward union of **14** and **22** was complicated by the unanticipated proclivity of **14** to polymerize under customary Michael conditions. The problem might have been solved by employing, for example, an α-substituted derivative of **14** less prone to polymerize [21,22], but this would have made an already elaborate route even lengthier. Heterogeneous catalysis provided a pleasantly simple solution. Thus, stirring a mixture of **14** and **22** in ethyl acetate containing suspended powdered Li_2_CO_3_ resulted in the nearly quantitative formation of **23** (Scheme 4). This diketone is a sensitive compound that was best advanced through the sequence in crude form. Treatment with NH_4_OAc in EtOH afforded dihydropyridine **23** (mixture of isomers), which, in crude form, was titrated in with a toluene solution of DDQ, whereupon pyridine **7** emerged with an 89% yield after chromatography over the 3-step sequence [19,23,24].

**Scheme 5:**
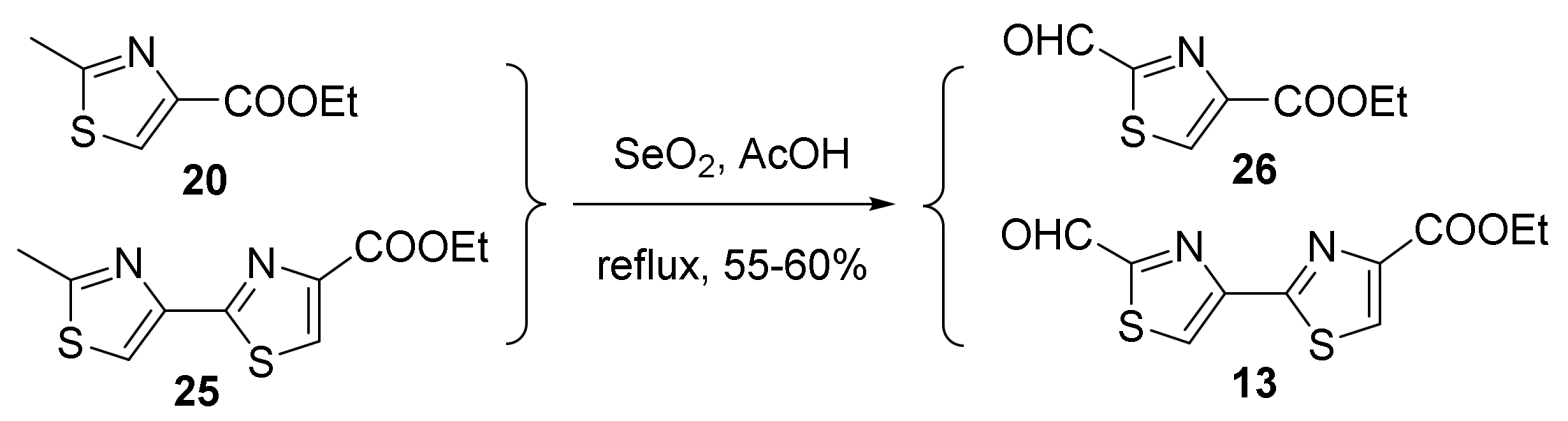
Oxidation of 2-methylthiazoles to the corresponding aldehydes.

Synthetic work toward thiocillin I provided an opportunity to refine the avenue to thiopeptidic heterocyclic clusters. First, a better synthesis of thiazole-2-carboxaldehydes was clearly needed. A plausible solution entailed a SeO_2_ oxidation of readily available 2-methyloxazoles. However, such a transformation appeared to be unknow. Indeed, it was determined that 2-methylthiazoles such as **20** and **25** were inert toward SeO_2_ in refluxing ethanol or dioxane (customary conditions), but they were rapidly converted into aldehydes **26** and **13** in refluxing acetic acid (Scheme 5). Some degree of overoxidation to a carboxylic acid and consequent decarboxylation was also observed, but the aldehydes were obtained with a 55–60% yield. A significantly more concise and efficient route had thus materialized [25].

**Scheme 6:**
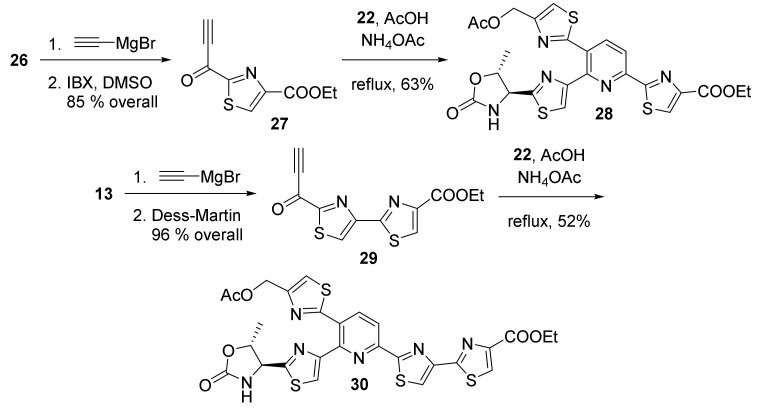
Bohlmann-Rahts avenue to pyridine **30**.

Second, a more direct method for creation of the pyridine ring was desirable. Important work by Moody [4], Bagley [26,27], and collaborators induced us to focus on a Bohlmann–Rahtz [28,29] approach. Ynones **27** and **28** were efficiently obtained from aldehydes **26** and **13**. Furthermore, it was found that their merger with segment **22** was attainable in one step, albeit in moderate yield, simply by refluxing in acetic acid solution in the presence of NH_4_OAc (Scheme 6). This transformation is a key step in the total synthesis of thiocillin I [30].

An awkward difficulty overshadowed the new procedure: the silyl group on segment **22** was lost upon refluxing in AcOH, and the alcohol thus liberated was converted into an acetate ester, probably through Fischer esterification (cf. **30** vs. **7**). It was not practical to advance **30** to a micrococcin or other thiopeptides, because the acetyl group would have interfered with subsequent manipulations. At that time, it was deemed best to convert **30** into **7**, even though this added two steps to the synthesis.

**Scheme 7:**
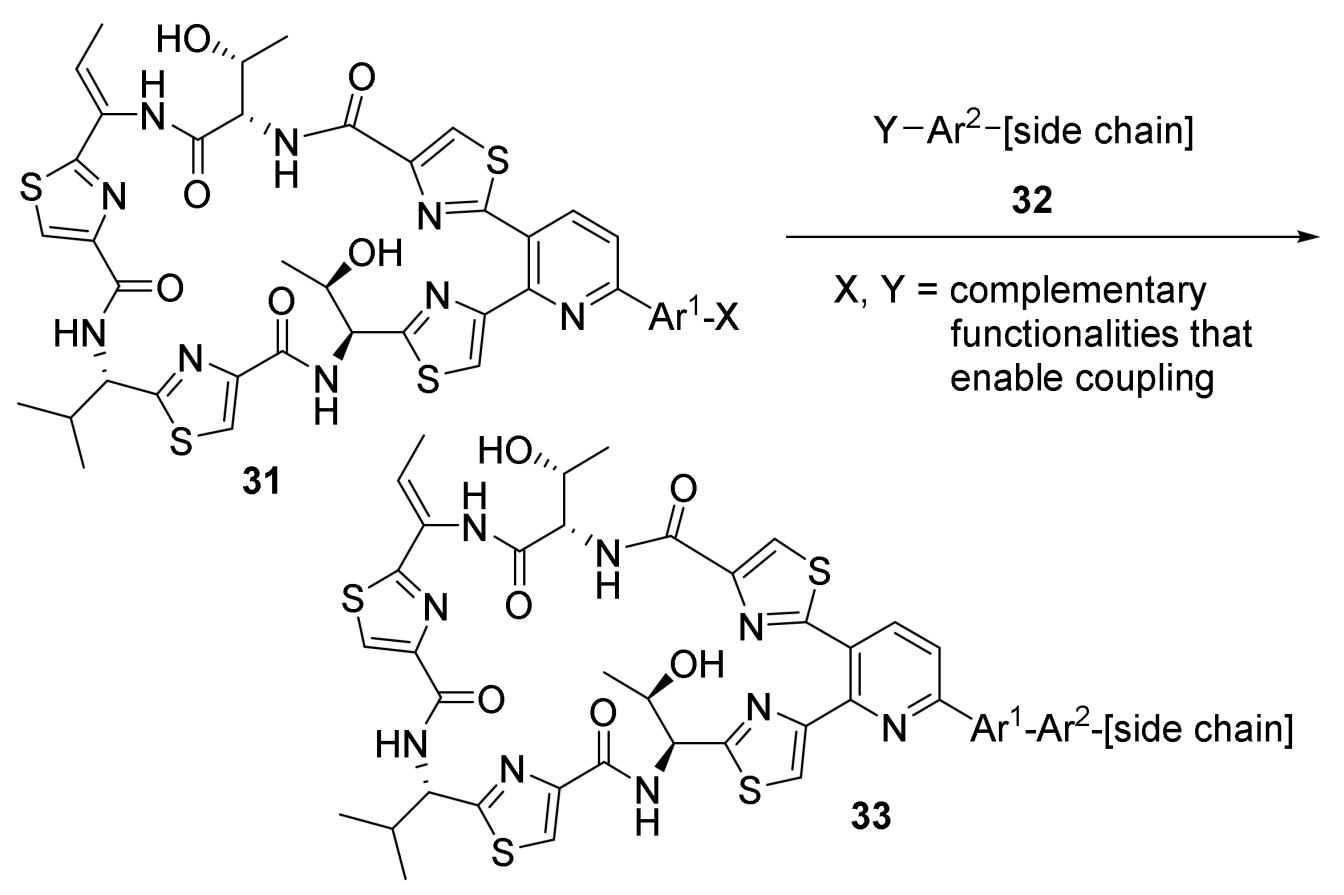
Hypothetical Suzuki avenue to micrococcin analogues **33**.

A “third-generation” approach to thiopeptide cores addressed the foregoing problem as well as the following issues. First, the silyl-protected primary alcohol in **22** and **7** derives from the carbethoxy group in **20** via reduction and silylation. Yet, at some point, the alcohol in question must be reoxidized to a carboxylic acid. A way to avoid such circuitous redox maneuvers was desirable. Second, early medicinal chemistry work with fully synthetic micrococcins and congeners had revealed that modifications of the macrocyclic ring led to loss of activity, while considerable latitude could be exercised at the level of the short side chain. Thus, an ideal new route would grant access to analogues exhibiting diverse side chains. Additional considerations induced us to research a strategy that entails the union of a fully formed macrocyclic segment with an aromatic moiety carrying a complete side chain (Scheme 7). Ease of scalability induced us to create two crucial thiazole rings using the White–Siegel thiazole synthesis [31], while the actual joining of fragments such as **31** and **32** was hoped to be achievable through a Suzuki coupling reaction [32].

**Scheme 8:**
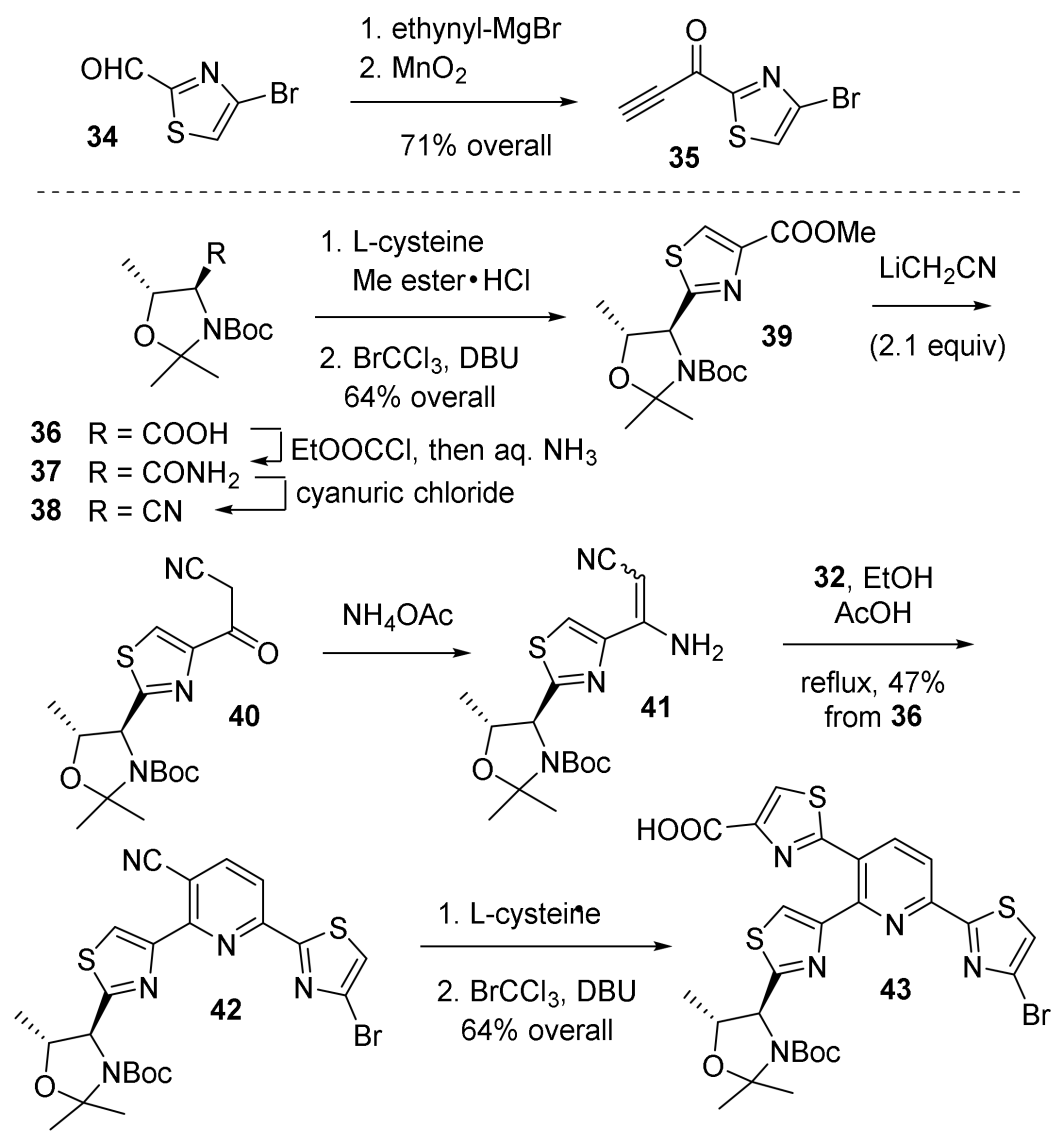
White-Siegel thiazole synthesis en route to pyridine **43**.

The assembly of a suitable form of macrocycle **31** is exemplified in Scheme 8 with the Bohlmann–Rahtz synthesis of **43**. Commercial educts **34** and **36** were advanced to ynone **35** and oxazolidine **39**, respectively. A minor inconvenience encountered on the way to **39** was that the conversion of amide **37** to the corresponding thioamide (required for the introduction of the thiazole), e.g., with the Lawesson reagent, was problematic, giving rise to an impure product that was difficult to purify. In contrast, dehydration of **37** to nitrile **38** and subsequent White–Siegel thiazole construction performed quite well. The resulting **39** lacks the relatively acidic N-H bond present in **19**. This allowed the use of only two equivalents of an organometallic agent, instead of three (cf. Scheme 3), in the reaction leading to its transformation into a suitable Bohlmann–Rahtz partner. Furthermore, it became apparent that effecting the condensation of **39** with lithiated acetonitrile, rather than lithiated **21** (Scheme 3), was advantageous for ease of purification. Indeed, ketonitrile **30** needed no purification at all, and it was advanced to pyridine **42** in 47% overall yield from commercial **36**. A final White–Siegel thiazole-forming reaction converted **42** into **43**.

**Scheme 9:**
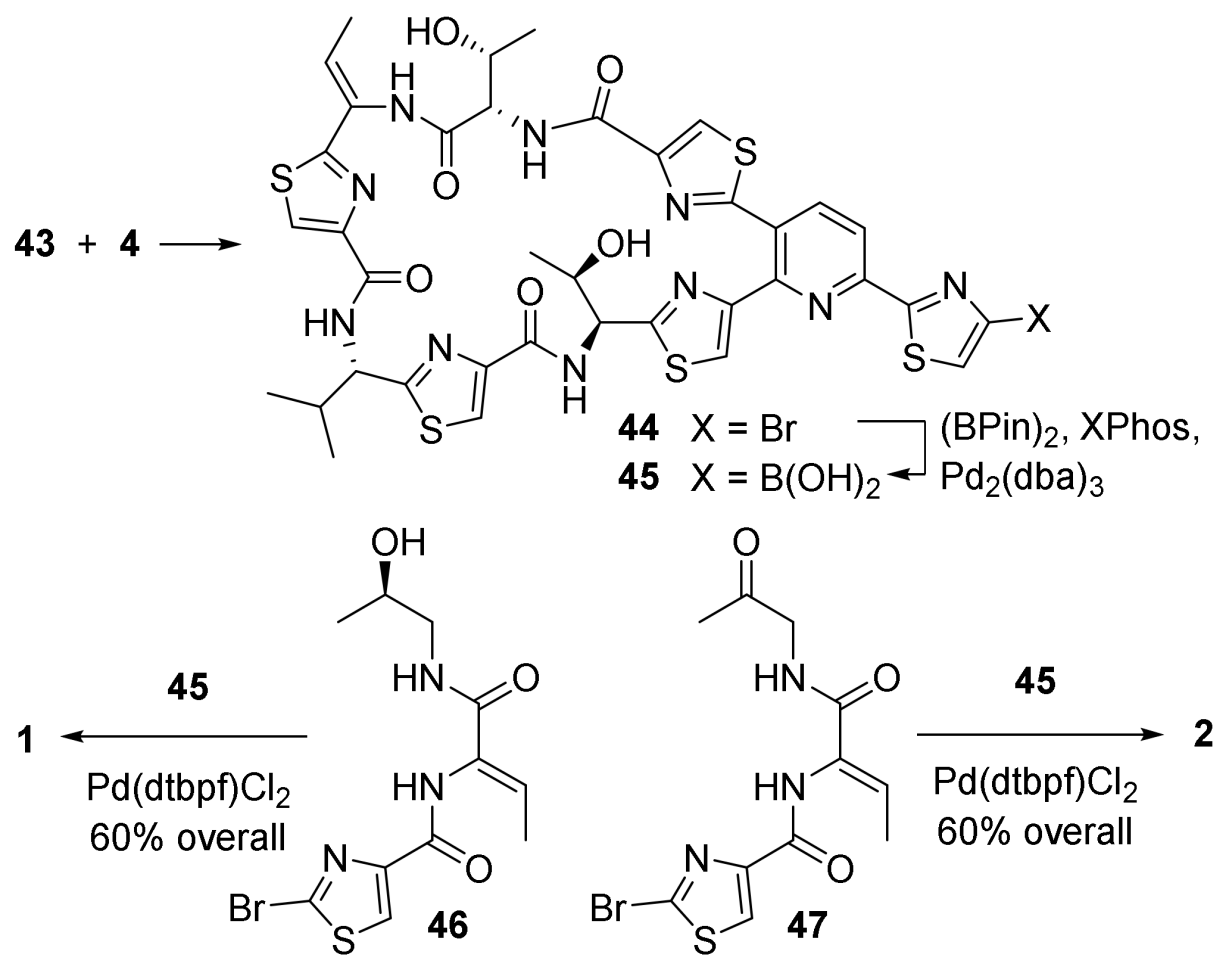
Synthesis of micrococcins P1 and P2 by Suzuki coupling.

The union of **43** with fragment **4** proceeded uneventfully to yield macrocycle **44**. To our knowledge, no examples of transition metal-mediated coupling reactions with substrates so densely packed with potential ligation sites for transition metals had been recorded in the literature prior to our work. This, of course, created significant uncertainty about the feasibility of the planned transformations. Fortunately, it rapidly transpired that **44** was an excellent substrate for Suzuki coupling with diverse boronic acids, and it was even amenable to conversion into boronic acid **45**, a sensitive material that was best employed in crude form [33]. The synthesis of micrococcins P1 and P2 from **45** was achieved in a single step by coupling with bromothiazoles **46** and **47**, respectively (Scheme 9). The preferred catalyst for this step was (di-*tert*-butylphosphino ferrocene)palladium(II)dichloride, which afforded slightly better yields than the more common Pd(PPh_3_)_4_ [33].

## 3. From Micrococcins to Masitinib^®^ and Beyond

In the midst of the campaign that led to the total synthesis of MP1, the senior author established a collaboration with AFIRMM, a French association of patients suffering from a rare genetic disease called mastocytosis [34]. This condition is associated with the aberrant activity of particular granulocytes termed mast cells, which are implicated in many pathologies [35]. Specifically, mastocytosis and other mast cell-related ailments are characterized by the unregulated activity of a mutated kinase termed c-kit, inhibition of which should thus contain or suppress the symptoms of such diseases. The objective of the collaboration was indeed the development of a c-kit inhibitor suitable for the treatment of mastocytosis.

**Scheme 10:**
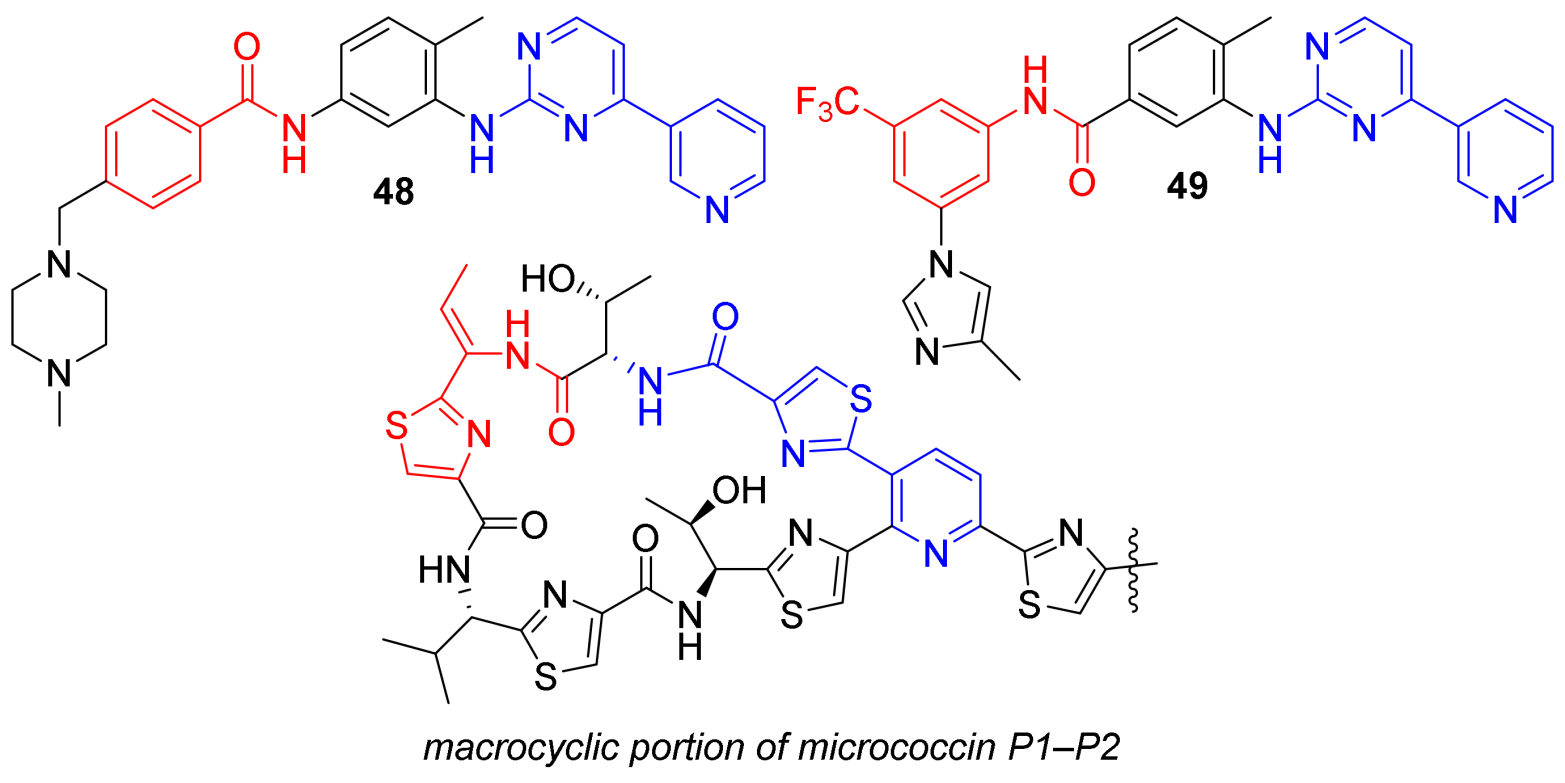
Structural similarities between kinase inhibitors and micrococcins.

**Scheme 11:**
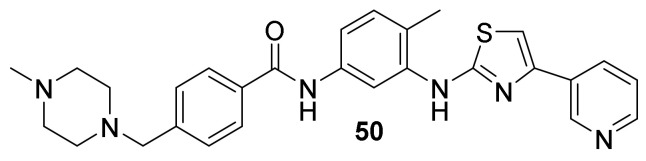
Structure of Masitinib^®^.

There is a striking structural similarity between certain kinase inhibitors, such as Imatinib^®^, **48** and Nilotinib^®^, **49** [36], and portions of the macrocycle of micrococcins P1–P2 (Scheme 10). Furthermore, it was known that **48**, originally optimized as an inhibitor of another mutant kinase associated with various cancerous diseases, such as chronic myelogenous leukemia (CML) and acute lymphocytic leukemia (ALL), and known as bcr-Abelson kinase, possessed off-target activity against c-kit. This inspired the exploration of structural hybrids of kinase inhibitors and thiopeptides with the intention of amplifying c-kit inhibitory activity. A few iterations led to a potent (low nM), highly selective inhibitor that was especially active against the Δ27 mutant of c-kit. This kinase is associated with gastrointestinal stromal tumor in humans and malignant mast cell tumor in canines. The compound became known as Masitinib^®^, **50** (Scheme 11) [37], now a product of AB Science, SA, Paris, France [38]. Interestingly, **50** is inactive against the bcr-Abelson kinase. In 2010, masitinib was approved in the European Union for veterinary applications, and it is currently in advanced Phase III clinical trials for a number of human conditions [38].

**Scheme 12:**
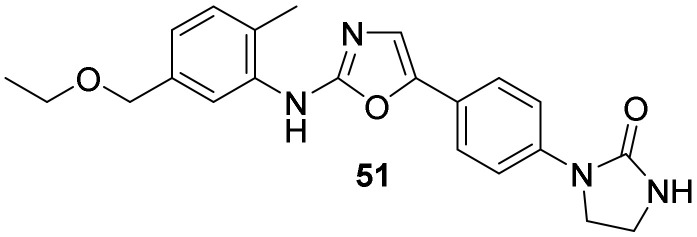
Structure of AB-8939.

Over the years, chemists at AB Science have produced thousands of masitinib analogues in an effort to identify inhibitors of potentially druggable kinases, thereby creating a large library of privileged structures. A screen of this library against various targets revealed that a compound nearly devoid of kinase inhibitory activity, AB-8939, **51** (Scheme 12), is instead a sub-nanomolar destabilizer of nanotubules, thus expressing colchicine-like activity. This descendant of masitinib is especially efficacious against particular blood cancers, and indeed, it is currently in clinical trials against acute myeloid leukemia [38].

**Scheme 13:**
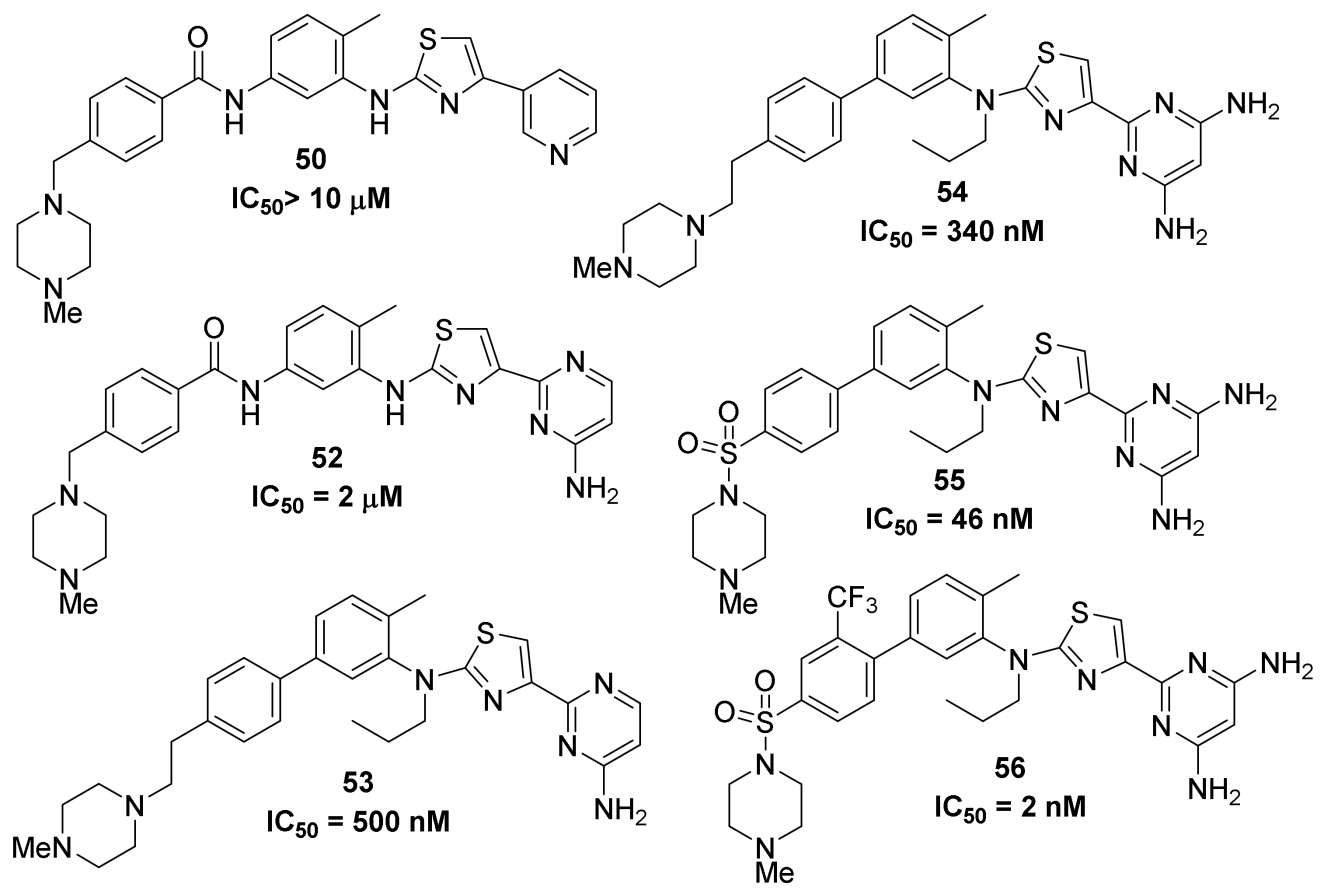
Structural evolution of masitinib into the potent dCK inhibitor, **56**.

Before long, it became apparent that masitinib possesses a number of other therapeutically valuable properties. Of note among these is its ability to enhance the potency of gemcitabine in the treatment of pancreatic cancer. A seminal study unveiled the remarkable mechanism through which this happens [39]. In the course of that work, it was also found that masitinib is a weak (>10 μM) inhibitor of 2′-desoxycytidine kinase (dCK). This enzyme controls the rate-limiting event of the nucleotide salvage pathway, and it has recently emerged as a prime target in cancer therapy. Provocative implications of that discovery induced distinguished colleagues at the Paoli-Calmettes Institute of Cancerology in Marseille, France, to launch a collaboration with the senior author with the aim of developing a masitininb analogue that would function as a more potent dCK inhibitor. Indeed, much discussion had led to the conclusion that a clinically useful inhibitor would have to have an IC50 below 10 nM. It was thus necessary to enhance the anti-dCK potency of masitinib almost 10,000 fold! A medicinal chemistry effort relying on advanced computational, crystallographic, and calorimetric methods, as well as a great deal of sophisticated organic synthesis, transmogrified masitinib into **56**, a 2 nM dCK inhibitor with appropriate pharmacological, toxicity, and selectivity properties [40]. The progression from **50** to **56** is summarized in Scheme 13. Compound **56** is now being readied for clinical tests.

Finally, toward the end of the maelstrom caused by the COVID-19 pandemic, a noteworthy paper in the August 19, 2021 issue of Science disclosed that masitinib is a broad coronavirus inhibitor that blocks the replication of SARS-CoV-2 [41].

The foregoing development does not constitute the final gift from the micrococcin cornucopia. Recall that **1**–**3** are potent antibacterials. The rapid pace at which microorganisms are developing antimicrobial resistance (AMR) is an alarming threat that is already causing many fatalities [42]. As a consequence, there is an urgent need to identify more effective antibiotics with new modes of action [43]. Thiopeptides appear to be excellent platforms for generating new anti-infective resources based on the foregoing criterion. Relevant discussion is part of the next section.

## 4. New Micrococcin-Based Antibiotics

Thiopeptide antibiotics are difficult to derivatize, possess low aqueous solubility, and are poorly absorbed from the gastrointestinal (GI) tract. This clearly handicaps the development of orally administrated, systemic anti-infective agents based on those natural products, which have thus remained largely unexploited as sources of new antibiotics. Yet, such weaknesses can be turned to advantage in particular circumstances. A case in point is GI infection with the Gram-positive bacterium, *Clostridioides difficile*. This organism is on the CDC list of urgent microbial threats [18]. More than 12,800 *C. difficile* deaths were reported in 2019 in the U.S. alone. Vancomycin and fidaxomicin are the only two antibacterials currently available to treat such infections. However, they are associated with recurrence rates of 15–30% and display much reduced efficacy against the emerging hypervirulent strain, *C. difficile* ribotype 027 [44]. There is thus an unmet medical need for new agents that are active against new strains and reduce recurrence rates.

*C. difficile* proliferates in the GI tract, especially the large intestine. An ideal antibiotic against that organism should be orally administrable yet be poorly bioavailable in order to target *C. difficile* in the gut. Furthermore, it should exert no untoward effects on beneficial intestinal microbiota, so as to reduce relapse. A noteworthy application of these principles is apparent in Novartis’ development of LFF571 against *Clostridioides difficile* infection (CDI). This semisynthetic antibiotic is derived from the natural thiopeptide, GE2270A, which is characterized by a 29-membered ring macrocycle [45,46,47].

**Figure 1:**
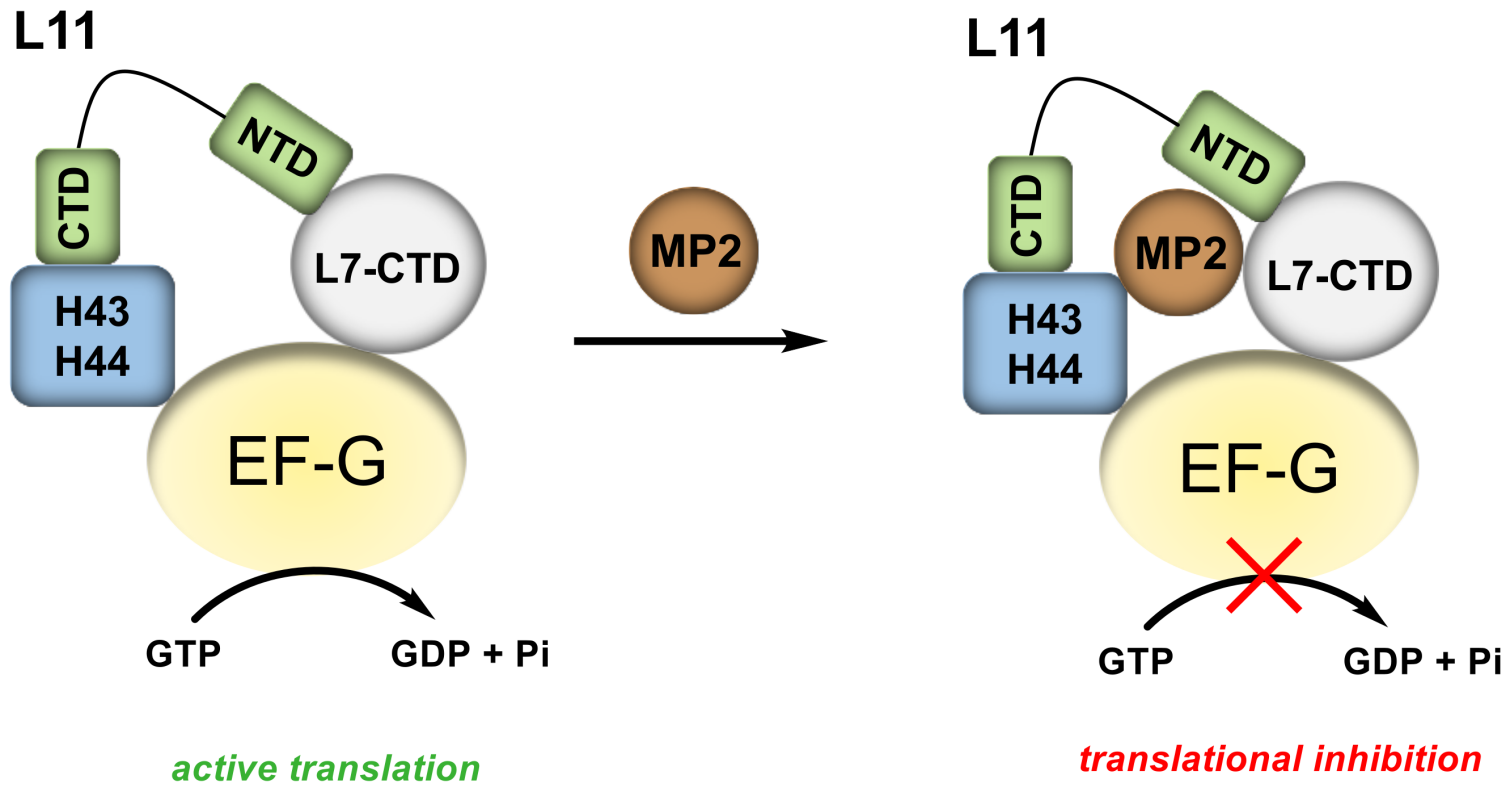
Schematic representation of the mode of action of micrococcin P2. (**Left**): the C-terminal domain (CTD) of L11 interacts with helices 43 and 44 of the 23s rRNA, and the N-terminal domain interacts with L7. EF-G is positioned between these two regions to convert GTP to GDP for protein translation to take effect. (**Right**): upon binding of MP2 to the region between L11 and 23s rRNA, the proper alignment for protein translation is disrupted, resulting in the inhibition of protein synthesis. NTD = N-terminal domain; CTD = C-terminal domain; L7 = riboromal protein L7; L11 = ribosomal protein L11; EF-G = Elongation factor G; MP2 = Micrococcin P2.

A visionary company in the Republic of Korea, A&J Science, Ltd., has invested significantly in research aiming to develop chemotherapeutic resources against *C. difficile* and other Gram+ microorganisms based on 26-membered thiopeptides such as micrococcins P1–P2. The unusual mechanism of action of these substances has been studied extensively [48,49]. They are believed to bind to a cleft of the bacterial ribosome located between the 23s rRNA and L11 domain [50,51]. As a result, the translational process of protein synthesis is blocked, ultimately resulting in the death of the microorganism (Figure 1).

**Figure 2:**
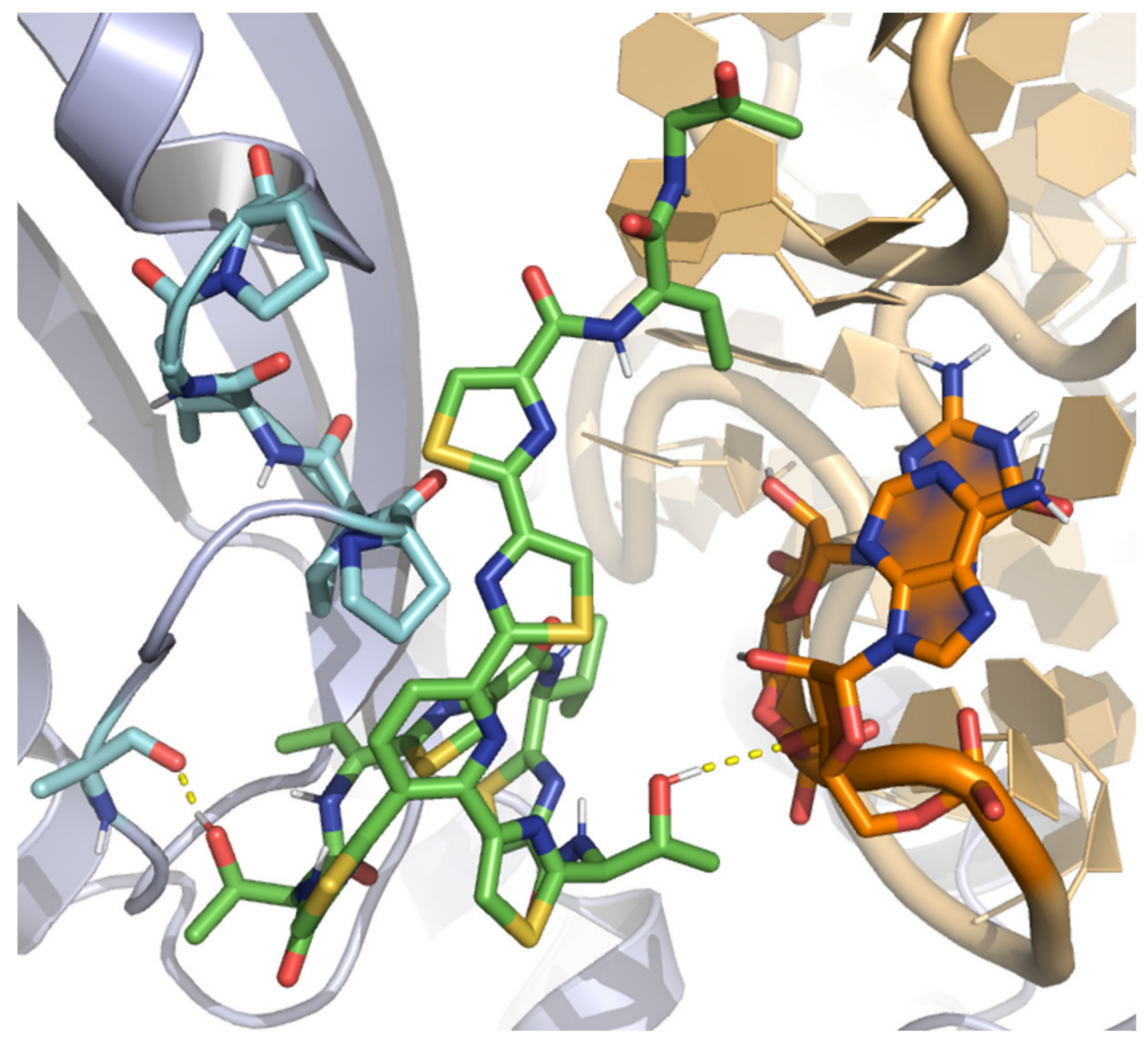

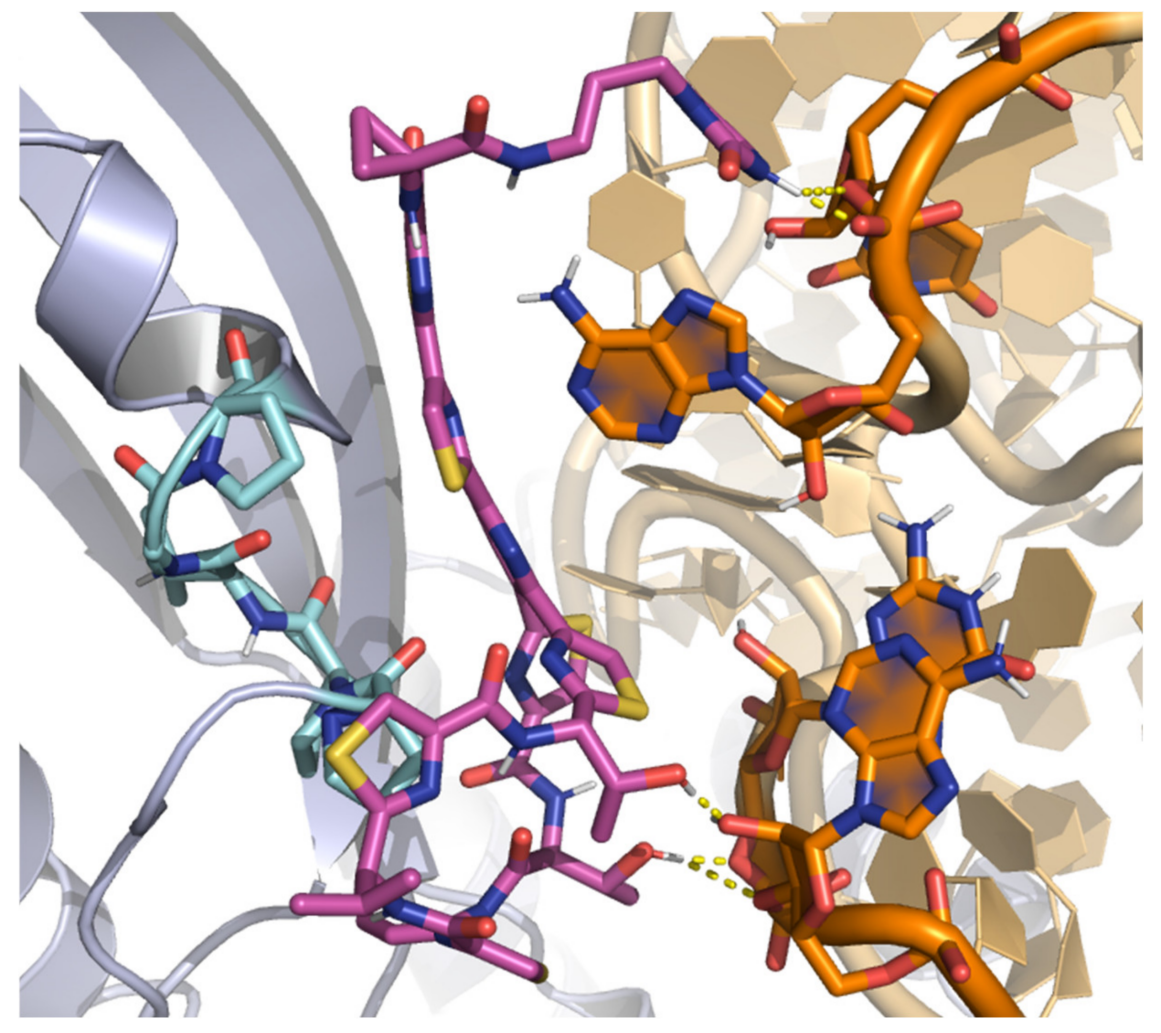
Best docking pose of MP2 and analogue 57 bound to their known receptor. L11 and 23s rRNA are represented by light blue and light orange ribbons, respectively. Interacting residues are shown as stick representations. Hydrogen bonds are shown as yellow dashed lines. (**Top**): computed binding pose of MP2 (green). (**Bottom**): computed binding pose of **57** (purple), showing additional hydrogen bonding interactions [54].

**Scheme 14:**
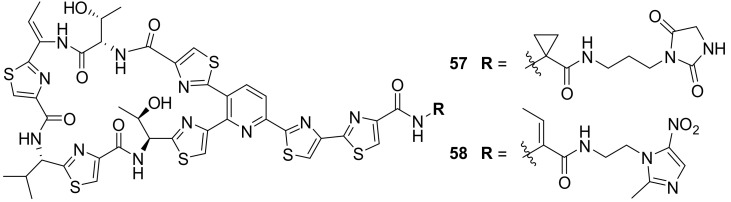
Structures of micrococcin analogues **57** and **58** with enhanced antibiotic potency.

The advent of an efficient synthesis of micrococcins enabled a medicinal chemistry campaign that rapidly established the efficacy of **1**–**2** vs. CDI, even against the problematic ribotype 027, without cross-resistance to existing antibiotics. Furthermore, **1**–**2** were found to possess suitable pharmacokinetic properties and not harm beneficial gut biota [52]. As briefly indicated in the introduction, structure–activity relationship (SAR) work with fully synthetic materials revealed that modifications/substitutions are tolerated only on the “eastern” side chain of micrococcins. Extensive computational simulations of the binding of **1**–**2** and congeners to their known receptor [51] (Figure 2) guided the creation of a library of analogues that were prepared by the chemistry of Scheme 9. This led to the discovery of compounds **57** and **58**, either of which is almost an order of magnitude more potent than vancomycin (Scheme 14 and Table 1) [53]. Notice that micrococcin P2 is equivalent to vancomycin against *C. difficile* and that **58** comprises a metronidazole-like subunit believed, inter alia, to enhance binding to its ribosomal receptor.

Compound **58** proved to be more efficacious than vancomycin and fidaxomicin in a mouse model of *C. difficile* ribotype 027 infection, both in terms of overall survival rate and recurrence [54]. The latter property was attributable to the preservation of a good consortium of gut microbiome, as determined by 16s rRNA sequencing [54]. In addition, extensive toxicological studies determined that **58** had an excellent safety profile. Accordingly, it was advanced to pre-clinical development.

**Scheme 15:**
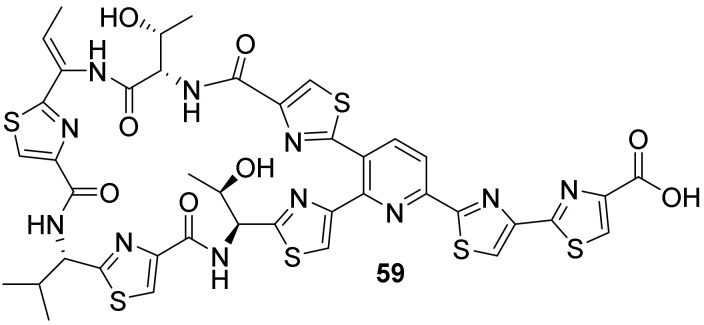
Structure of micrococcin acid.

**Scheme 16:**
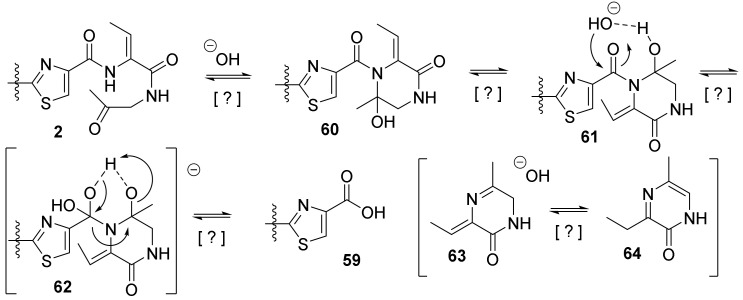
Hypothetical (hence the [?] symbol) mechanism of hydrolysis of MP2 to **59**.

The structures of **57** and **58** reflect the fact that many chemotherapeutically useful analogues of **1**–**2** are amides of what may be termed “micrococcin acid”, **59** (Scheme 15). While the chemistry of Scheme 3, Scheme 4, Scheme 5, Scheme 6, Scheme 7, Scheme 8 and Scheme 9 can certainly provide quantities of this material, it would be desirable to produce it biosynthetically. This would greatly facilitate access to semisynthetic micrococcin congeners. The biosynthetic pathway of micococcins would be difficult to modify so as to produce **59** selectively. However, it should be easily alterable to afford virtually only MP2, in that MP1 is derived from MP2 though the action of a ketone reductase. Suppression of the latter halts the biosynthetic process at the stage of **2**. Furthermore, the presence of a ketone in MP2 led to the hypothesis that selective base hydrolysis to **59** might be possible through the mechanism adumbrated in Scheme 16. Briefly, it seemed likely that the action of base upon **2** would promote equilibration with cyclic hemiamidal **60**. The OH group in **60** could now direct a nucleophilic attack of the hydroxide ion onto the correct amide carbonyl through hydrogen bonding (cf. **61**). Tetrahedral intermediate **62** seemed primed to undergo entropically driven fragmentation to acid **59**, compound **63**, and hydroxide ion. The acid, rapidly converted into a salt under the basic conditions of the reaction, would be retrieved following acidification. The fact that **63** can easily tautomerize to aromatic pyridazinone **64** under basic conditions seems to contribute additional driving force to the process.

**Scheme 17:**
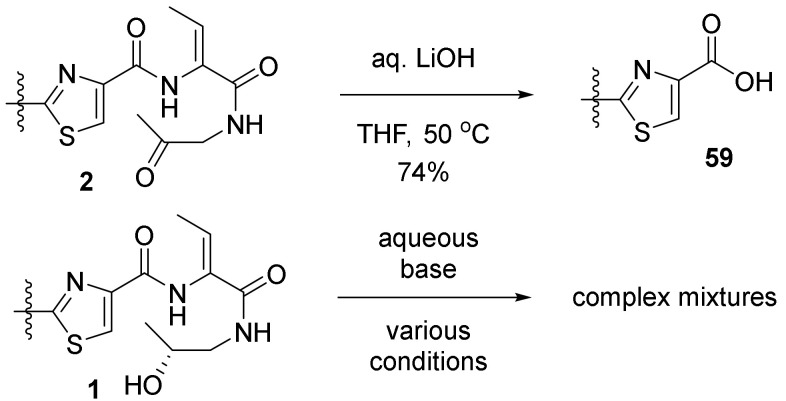
Dichotomous behavior of MP2 and MP1 toward base hydrolysis.

In the end, it transpired that brief treatment of MP2 with aqueous LiOH in THF at 50 °C, followed by acidification, cleanly produces acid **59** in 74% yield [33]. No effort was made to isolate **63** or **64** from the hydrolysis mixtures. In contrast, treatment of MP1 under a multitude of basic conditions returns intractable mixtures of products containing little or no **59** (Scheme 17) [33].

On the basis of the foregoing, a molecular biology effort aiming to ferment only micrococcin P2 was launched. This led to the successful creation of a mutant *B. subtilis* that produces only **2** [33]. Ongoing efforts strive to optimize the biosynthetic route to **2**, and consequent access to **59**, opening the door to a number of new antibacterial resources that might enable chemotherapeutic intervention against many serious microbial threats.

## 5. Epilogue

The reader may appreciate that the above contributions to the biomedical field stemmed from what initially seemed a purely academic, curiosity-driven endeavor, i.e., the total synthesis of thiopeptides. Indeed, the field of academic natural product synthesis has provided (and continues to provide!) many important leads and solutions to pressing medical needs. Suffice it to cite, in addition to the foregoing, Eribulin^®^ and Ixabepilone^®^, which emanated from synthetic efforts toward halichondrin B and epothilone, respectively. Yet, natural product synthesis has become a badly neglected—some would say beleaguered—area of research. The authors hope that this state of affairs will soon be righted, and that in the not-too-distant future, chemistry departments and funding agencies alike will see it fit to resume investment in natural product synthesis: a science as central as ever to any effort to improve human health.

## Figures and Tables

**Table 1:** Minimum inhibitory concentration (MIC, μg/mL) of vancomycin, MP2, **57**, and **58**, against *C. difficile* ATTC 43325.

Antibiotic	MIC (μg/mL)
vancomycin	1.0
**2**	1.0
**57**	0.125
**58**	0.125

## Data Availability

Please consult the citations to original articles for available data or contact the authors. Certain data may be unavailable because of IP and privacy issues.
